# Intracardiac radiofrequency ablation in living swine guided by polarization-sensitive optical coherence tomography

**DOI:** 10.1117/1.JBO.25.5.056001

**Published:** 2020-05-08

**Authors:** Xiaowei Zhao, Orhan Kilinc, Colin J. Blumenthal, Deniz Dosluoglu, Michael W. Jenkins, Christopher S. Snyder, Mauricio Arruda, Andrew M. Rollins

**Affiliations:** a Case Western Reserve University, Department of Biomedical Engineering, Cleveland, United States; b Rainbow Babies and Children’s Hospital, The Congenital Heart Collaborative, Cleveland, United States; c Case Western Reserve University, School of Medicine, Cleveland, United States; d Case Western Reserve University, Department of Electric Engineering and Computer Science, Cleveland, United States; e Case Western Reserve University, Department of Pediatrics, Cleveland, United States; f University Hospitals Cleveland Medical Center, EP Laboratories and the Atrial Fibrillation Center at the Harrington Heart and Vascular Institute, Cleveland, United States

**Keywords:** polarization-sensitive optical coherence tomography, radiofrequency ablation, cardiac arrhythmia

## Abstract

**Significance:** Pulmonary vein isolation with catheter-based radiofrequency ablation (RFA) is carried out frequently to treat atrial fibrillation. However, RFA lesion creation is only guided by indirect information (e.g., temperature, impedance, and contact force), which may result in poor lesion quality (e.g., nontransmural) and can lead to reoccurrence or complications.

**Aim:** The feasibility of guiding intracardiac RFA with an integrated polarization-sensitive optical coherence tomography (PSOCT)-RFA catheter in the right atria (RA) of living swine is demonstrated.

**Approach**: In total, 12 sparse lesions were created in the RA of three living swine using an integrated PSOCT-RFA catheter with standard ablation protocol. PSOCT images were displayed in real time to guide catheter-tissue apposition. After experiments, post-processed PSOCT images were analyzed to assess lesion quality and were compared with triphenyltetrazolium chloride (TTC) lesion quality analysis.

**Results:** Five successful lesions identified with PSOCT images were all confirmed by TTC analysis. In two ablations, PSOCT imaging detected gas bubble formation, indicating overtreatment. Unsuccessful lesions observed with PSOCT imaging were confirmed by TTC analysis.

**Conclusions:** The results demonstrate that the PSOCT-RFA catheter provides real-time feedback to guide catheter-tissue apposition, monitor lesion quality, and possibly help avoid complications due to overtreatment, which may enable more effective and safer RFA treatment.

## 1 Introduction

Pulmonary vein (PV) isolation (PVI) with catheter-based radiofrequency ablation (RFA) is commonly performed to treat atrial fibrillation (AF).^1^ The goal of a PVI is to electrically disconnect arrhythmogenic PVs from the left atrium (LA). This is done using RFA to create circular lines of lesions that surround the PVs and isolate them from the LA.^2^ To achieve electrical isolation, lesion transmurality and lesion line continuity are crucial.^3^^–^^5^ Lesion formation is currently guided with indirect information (e.g., temperature, impedance, and contact force). However, PVI procedures still suffer from a high recurrence rate and complications due to several factors (e.g., nontransmural lesions, steam pops, perforation, and damage to the adjacent area from energy delivery).^3^^,^^4^^,^^6^ Currently, more than half of patients who receive RFA PVI therapy will require additional RFA procedures. Only 17% of patients are arrhythmia-free at 5 years after a single AF ablation procedure. Even with multiple procedures, 2.1 on average, the success rate is approximately 65% after 5 years.^7^ This suggests that direct tissue-measurement guidance may improve PVI efficacy.

M-mode optical coherence tomography (OCT) [i.e., optical coherence reflectometry (OCR)] has guided catheter-tissue apposition in *ex-vivo* experiments^8^ and monitored RFA in *ex-vivo* and *in-vivo* experiments.^9^^,^^10^ However, OCR has not been demonstrated to identify and differentiate tissue structure, which is potentially important for RFA guidance. Our previous *ex-vivo* and *in-vivo* experiments demonstrated that OCT imaging can guide and monitor cardiac RFA therapy in real time with direct tissue measurement.^11^^–^^13^ OCT can confirm catheter-tissue apposition,^11^ differentiate cardiac tissue types,^12^ and detect RFA lesion formation.^11^^,^^13^ Catheter-based, intracardiac OCT imaging via percutaneous access was demonstrated in living swine with a stand-alone OCT probe.^14^ Polarization-sensitive optical coherence tomography (PSOCT) was shown to provide superior contrast for detecting RFA lesion formation via a catheter probe.^15^ This is because PSOCT is sensitive to tissue birefringence, which is strongly reduced by thermal ablation such as RFA. Finally, a prototype integrated PSOCT-RFA catheter was developed and simultaneous RFA and real-time guidance with PSOCT was demonstrated *ex vivo* in excised swine right ventricular tissue.^16^ In this work, we demonstrate *in-vivo* monitoring of cardiac RFA lesion formation using integrated PSOCT-RFA catheter prototypes in the right atrium (RA) of living swine via percutaneous access under the guidance of single-plane fluoroscopy.

## 2 Methods

In this work, a portable PSOCT system that irradiates the sample with a single polarization state, as previously reported, was used.^16^ The axial resolution and sensitivity of this system is measured to be 10  μm [full width at half maximum (FWHM)] and 105 dB, respectively. Structure and net retardance images were calculated with the algorithm described by Hitzenberger et al.^17^ Net retardance is the accumulated effect of tissue birefringence on a beam of light over depth and is the common parameter used to visualize PSOCT images of birefringent tissue.^18^ The integrated PSOCT-RFA catheter was built based on a standard clinical 2.3 mm (7 Fr) RFA catheter (Blazer II HTD, Boston Scientific).^16^ To incorporate a 1-mm-diameter OCT probe while maintaining the RFA functionality, the steering cables in the catheter and the thermistor at the catheter tip were removed. The center lumen of the ablation electrode was enlarged and a 1-mm-diameter glass window was secured in the electrode tip to isolate blood and allow for forward-view imaging. Image transverse resolution at focus was 17  μm (FWHM). Images were acquired at a speed of 20  frames/s with 2000 A-scans per B-scan. For guidance of catheter-tissue apposition and observation of lesion formation for all ablation attempts, real-time display of the PSOCT structure and net retardance images were updated at a frame rate of 6  frames/sec (processed on a CPU and limited by current software speed). Raw data were saved at full speed (20  frames/sec) and analyzed in postprocessing for detailed lesion formation observation.

To evaluate the functionality of the integrated PSOCT-RFA catheter in a living heart, experiments were done in the RA of three healthy swine (domestic swine, weight 31±1  kg, female) in the Surgical Training and Research (STAR) Core at Case Western Reserve University. The study protocol was reviewed and approved by the Institutional Animal Care and Use Committee of Case Western Reserve University. The percutaneous catheterization procedure was conducted with the guidance of single-plane fluoroscopy. During the procedure, the swine was anesthetized, intubated, and mechanically ventilated. Because this integrated catheter prototype was not steerable, a steerable introducer with an 8.5-F internal diameter (St. Jude, Agilis) was advanced into the RA via the right femoral vein and was used to guide the integrated catheter. Since the thermistor was removed to accommodate the OCT probe, all lesions were made with the ablation generator (Maestro 3000, Boston Scientific) in power control mode with a fixed ablation protocol (8 to 15 W power, 300-ohm impedance limit, 50-s ablation time). Lesions were sparsely made in the RA with locations noted relative to anatomical landmarks (e.g., coronary sinus, superior vena cava, tricuspid valve, and ribs for superior and inferior locations) under the guidance of fluoroscopy. After the experiments, the pigs were sacrificed, and hearts were harvested, the RA was dissected, cut open, and stained with 1% triphenyltetrazolium chloride (TTC) phosphate-buffered saline solution at 37°C for 30 mins. With TTC staining, viable tissue is stained red, while RF lesions are white. Identified lesions were matched to corresponding PSOCT images by referring to the relative locations and to anatomical landmarks as recorded.

## 3 Results

**Fig. 1:**
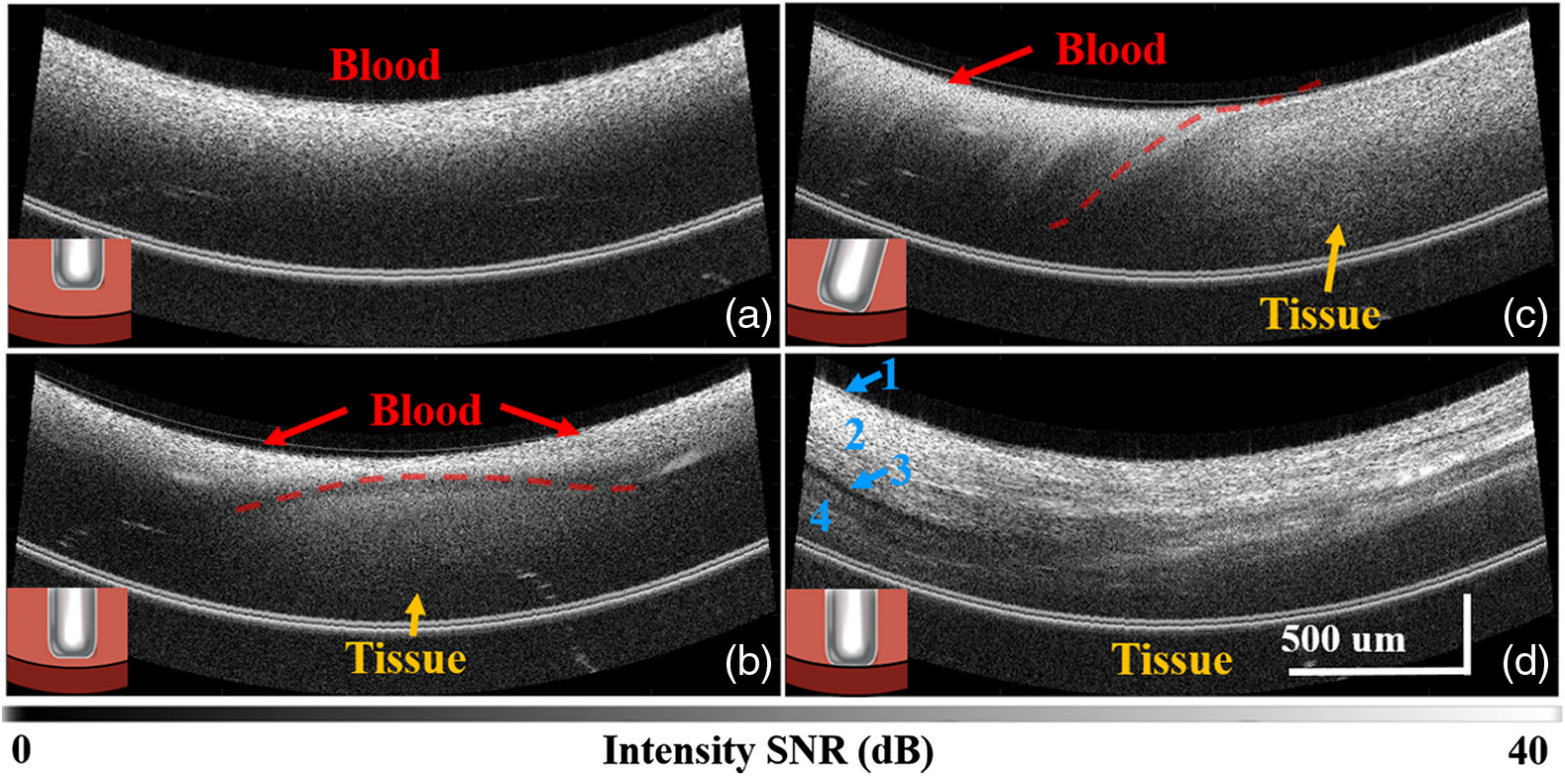
PSOCT intensity images guiding catheter-tissue apposition. The figure shows when the catheter is (a) not in contact, (b) approaching the tissue, (c) making contact at an angle, and (d) in perpendicular contact with the tissue. In (d), 1, 2, 3, and 4 indicate the endocardium, myocardium, epicardium, and pericardium, respectively.

In OCT images, blood and tissue have distinct features, which enables guidance of catheter-tissue contact. Blood appears as a homogenous granular medium with high scattering and attenuation, while cardiac tissue has detailed tissue structure and deeper imaging depth. Figure 1 shows OCT intensity images used to guide catheter-tissue contact. Figure 1(a) shows an OCT image while the catheter is not in contact with the tissue. As the catheter approaches the tissue, blood between the catheter and the tissue is displaced by the catheter and the tissue starts to appear in the image [Fig. 1(b)]. Figures 1(c) and 1(d) show that OCT imaging can be used to differentiate perpendicular and angled contact, which affects lesion shape and size during ablation. In this living heart, endocardium, myocardium, epicardium, and pericardium can be seen from top to bottom as indicated by 1 to 4, respectively, in Fig. 1(d).

**Fig. 2:**
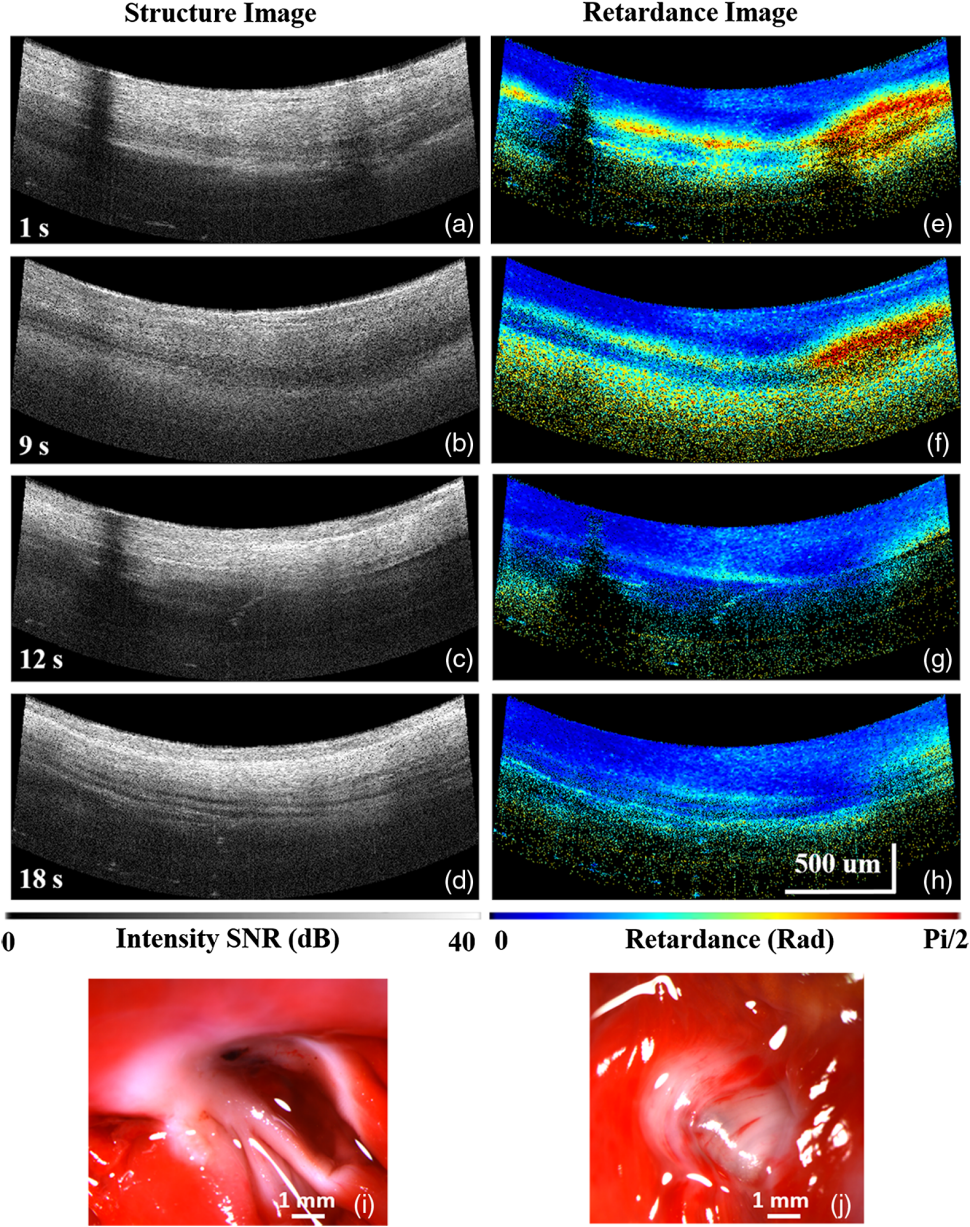
PSOCT monitoring of a transmural lesion created with 10 W power with the integrated catheter. (a)–(d) and (e)–(h) The structure images and corresponding net retardance images during lesion formation from 1 to 18 s. (i) and (j) Pictures of the endocardium and epicardium of the TTC stained lesion (Video 1, MP4, 10 MB [URL: https://doi.org/10.1117/1.JBO.25.5.056001.1]).

During ablation, PSOCT can provide contrast to monitor lesion formation in real time by tracking tissue birefringence changes. Cardiac tissue has strong birefringence due to well-aligned cardiomyocytes and subcellular anisotropy as a result of alignment of proteins within cardiomyocytes.^18^^–^^20^ An example of transmural lesion formation is shown in Fig. 2 (Video 1). Birefringence is displayed as colored bands in the calculated net retardance images. As the tissue is treated, tissue birefringence is lost and the colored bands disappear in net retardance images, as shown in Figs. 2(e)–2(h). At the same time, the tissue becomes more scattering and more homogeneous in the intensity images [Figs. 2(a)–2(d)]. The transmural lesion-formation time based on PSOCT images is defined as the time point after initiation of RF energy delivery when birefringence has been lost and does not change further. According to the OCT and PSOCT images in Figs. 2(c) and 2(g), the lesion has already formed transmurally by 12 s. Continuing ablation did not further change the PSOCT image of the tissue [Figs. 2(d) and 2(h)], and RF energy stop was triggered at 20 s due to high impedance. This result agrees with the TTC stain analysis, as shown in Figs. 2(i) and 2(j), which shows that the lesion was transmural.

**Fig. 3:**
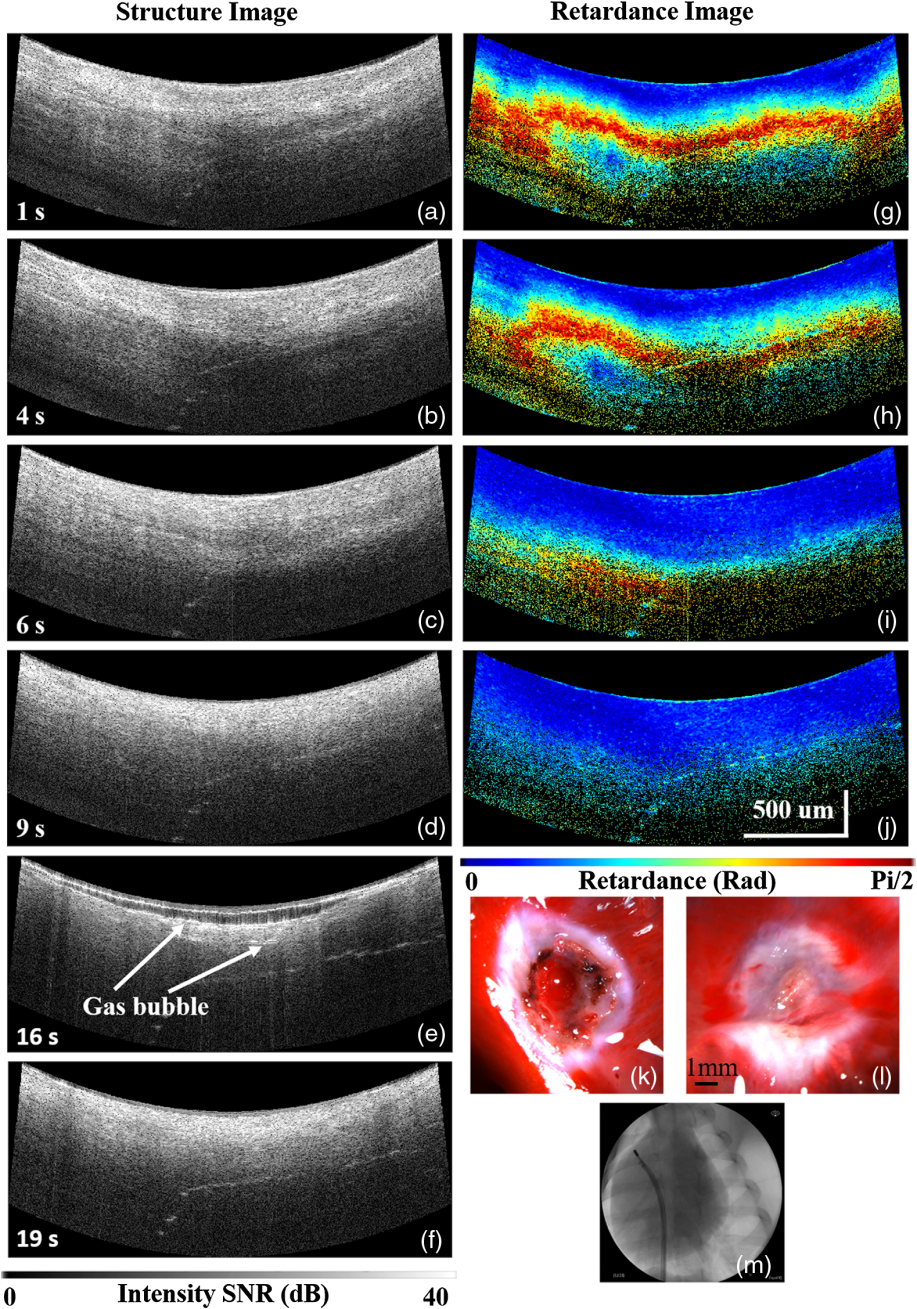
PSOCT monitoring of an overtreated lesion created with 10 W power with the integrated catheter. (a)–(d) and (g)–(j) The structure images and corresponding net retardance images during lesion formation from 1 to 9 s. With overtreatment, gas bubbles (e) and (f) formed and exploded, respectively, causing tissue damage. Pictures of the (k) endocardium, and (l) epicardium of the TTC stained lesion. (m) Screenshot of the single-plane fluoroscopy guidance (Video 2, MP4, 10 MB [URL: https://doi.org/10.1117/1.JBO.25.5.056001.2]).

Figure 3 (Video 2) shows a second example of a lesion created with the integrated PSOCT-RFA catheter. In this case, overtreatment is apparent. Similar to Fig. 2, an increase of scattering in the structure images [Figs. 3(a)–3(d)] and loss of the birefringence in the net retardance images [Figs. 3(g)–3(j)] through the cardiac wall indicate that this lesion was formed transmurally at 9 s. Continuing the ablation created gas bubbles in the tissue [Fig. 3(e)], which exploded (i.e., a steam pop), triggering the ablation generator to stop the energy delivery after an additional 3 s due to high tissue impedance [Fig. 3(f)]. After TTC staining, the endocardium and epicardium of this lesion [Figs. 3(k) and 3(l), respectively] indicate that it was transmural. But the endocardial surface was disrupted by overtreatment. Blood coagulation and char at the site of disruption can be seen as a red spot with a black edge at the center of the lesion.

In this study, 12 lesions were attempted in the RA of three swine. Results of all attempts are summarized in Table 1. Lesion quality verified by PSOCT images is compared with the gold standard of TTC staining. Five of the lesions were determined to be transmural by TTC. Four out of the five transmural lesions were detected with PSOCT imaging. The PSOCT data corresponding to one transmural lesion were of poor quality and tissue birefringence could not be detected. Two overtreated lesions were detected with PSOCT imaging by visualization of gas bubble formation. In one of these lesions, overtreated tissue was identified after TTC staining by identifying tissue surface disruption. However, because the other was in an area with significant trabeculation, tissue surface disruption was difficult to identify by inspecting after TTC staining. In the case of five attempted lesions, the catheter failed to deliver energy and both TTC staining and PSOCT confirmed that no lesions were formed. For all ablated lesions, with PSOCT monitoring, the transmural lesion-formation time was 9±4  s.

**Table 1:** Summary of PSOCT monitoring.

Category	Number	Contact guidance and lesion quality assessment			
		PSOCT monitoring		TTC stain	
Contact guidance for ablation	12	12		Not applicable	
Transmural lesion	5	4 transmural	1 poor polarization quality	5 transmural	
Overtreated lesion	2	2 overtreated		1 overtreated	1 ambiguous
Ablation failure	5	5 failure		5 failure	

## 4 Discussion

Catheter-tissue apposition is important because the contact area that the RF electrode makes with tissue strongly impacts lesion size and shape.^21^ In this study, all recorded lesions were made with perpendicular contact as determined by PSOCT imaging, as shown in Fig. 1.

Nontransmural lesions are believed to be one of the main causes of the high recurrence of AF.^4^^,^^5^ With PSOCT real-time monitoring, lesion quality may potentially be monitored directly to improve procedure efficacy. In this study, the loss of tissue birefringence between the endocardium and the epicardium, observed as the disappearance of colored bands in the net retardance images, and increased scattering in structure images (as shown in Fig. 2)^16^ were the image features used for evaluating transmural lesion formation. TTC staining of lesion target tissue after heart excision was used to confirm lesion quality. A lesion was considered to be transmural by TTC staining when the white-stained tissue could be seen from endocardium to epicardium, as shown in Figs. 2(k) and 2(l). Seven transmural lesions were completed in this study (two of them were overtreated). Of these, six were clearly identified as complete in PSOCT imaging.

One lesion was not successfully detected by PSOCT imaging due to the alignment of the polarization state of the imaging beam. For single-incident polarization state PSOCT, which was used in this study, the imaging beam should be circularly polarized for optimum contrast and accuracy. However, this is difficult to control because the polarization state of the beam changes with catheter manipulation and probe rotation. In these experiments, the polarization state of the system was optimized before the procedures and not for each single lesion during procedure. This impact can be reduced by extracting polarization state change from the tissue surface and compensating through the depth. Nevertheless, when the imaging beam is linearly polarized parallel or perpendicular to tissue fiber orientation, tissue birefringence is not detected. This is apparently what happened in this failed case. Tissue birefringence was not detected before ablation, so the expected change could not be detected. To overcome this limitation, future studies will make use of PSOCT technologies that illuminate the tissue with two orthogonal incident polarization states. These are more robust against rotation and allow for a more reliable measurement of tissue birefringence.^22^^,^^23^

Swine are commonly used as animal models to study RFA technologies because the swine heart has similar anatomy to the human heart,^24^^,^^25^ and pigs around 70 to 80 kg in weight have similar LA and RA wall thickness as adult human hearts.^26^ The study presented here made use of animals procured for another purpose (intracoronary OCT training) to fully use the animal, and the pigs were young and small (heart mass 192+/−15g). The RA wall at all 12 lesion sites was thin and fully captured within the field of view of the OCT imaging (wall thickness 655+/−525  μm). It is likely that the atrial wall will be too thick in some locations of larger hearts to be imaged transmurally by OCT. However, recent studies of adult clinical patients’ LA wall thicknesses with high resolution (0.31 to 0.5  mm/pixel) cardiac computed tomography has shown that the thickness of the majority of the LA wall, especially the posterior wall, is less than 2 mm.^27^^,^^28^ The wall will be even thinner under catheter pressure during ablation. Therefore, OCT has the potential to monitor RFA in the LA of human patients. Future studies will examine RA and LA wall thickness in living hearts of various sizes to better understand this potential variability and how it may limit the monitoring of atrial RFA by PSOCT.

A steam pop is an audible intramural gas bubble explosion caused by overtreatment during ablation.^29^ It damages the cardiac wall, exposes the underlying myocardium, creates tissue debris, and may lead to perforation or thrombosis.^30^ It can also create char on the ablation electrode, which reduces ablation efficiency. Although steam pops are generally defined by the audibility of the complication, silent steam pops have been observed with intracardiac echocardiography (ICE).^31^ The risk of steam pops makes it even more difficult to balance creating high-quality RFA lesions and preventing overtreatment in real time. In this experiment, two steam pops occurred. In both cases, gas bubbles within the myocardium were observed by PSOCT over 4 s before eruption. If the RF energy were stopped after these subtle precursors were detected, the steam pop could be avoided, thus improving the safety of the procedure by reducing the risk of this serious potential complication.

In this study, five of the twelve attempted lesions failed to form. In each case, both PSOCT image monitoring and TTC staining confirmed that no thermal lesion formed at the location. While these cases served as a useful negative control, they also revealed a catheter design flaw. In all five cases, both real-time PSOCT imaging and impedance measurements (about 110 ohm) indicated that the catheter was in contact with tissue. However, high impedance (over 300 ohm) developed within 10 s and automatically stopped the ablation. Furthermore, blood coagulation was found around the electrode after these attempts. These lesion failures were the result of limited contact area between the ablation electrode and tissue. When the Blazer II RFA catheter was modified to incorporate PSOCT imaging, the center lumen of the ablation electrode was enlarged, leaving the electrode tip with a 0.6-mm-thick ring around a 1-mm-diameter glass window. In addition, the ablation electrode has a dome-shaped tip and the window glass is flush with the electrode surface. Therefore, when PSOCT indicated that the window glass was in perpendicular contact with the tissue, it was still possible for the ablation electrode to be in poor contact with tissue, especially with the heart wall under tension. In future prototypes, we will enlarge the ablation electrode contact area. At the same time, we will slightly recess the window glass surface relative to the electrode contact surface, so tissue contact with the window glass will ensure contact between the ablation electrode and tissue.

While this work demonstrates the guidance functionality of the PSOCT-RFA catheter in percutaneous RF procedures in a setting similar to a clinical electrophysiology scenario, this study was limited to the RA of living swine instead of the LA, where PVI is conducted. Although tissue structure in the LA around PVs is different from the RA, tissue scattering and birefringence change during ablation are similar.^32^ Therefore, these results serve as a proof of concept for using PSOCT to guide RFA and justify future studies in the LA. In the future, *ex-vivo* and *in-vivo* experiments will be conducted to validate the transmural lesion formation time by stopping ablation at the time of loss of tissue birefringence, as determined by real-time PSOCT. To test a PSOCT-RFA catheter in the LA, future living animal studies will be carried out in a lab outfitted with a biplane fluoroscopy, ICE, and 3D mapping to perform a transseptal puncture and map the experiment.

## 5 Conclusion and Future Directions

This work demonstrates the basic functionality of a PSOCT-RFA catheter in a mock clinical setting. Results demonstrate that PSOCT imaging can guide catheter-tissue apposition, monitor lesion formation, and detect overtreatment in real time in living swine. In future work, we will improve the catheter design by incorporating temperature measurement, electrogram recording, and compatibility with a 3D mapping system. This will enable testing of the technology in more realistic procedures, including introducing the integrated catheter into the LA via transeptal puncture to simulate a PVI. We will also improve the PSOCT system using two orthogonal illumination polarization states to improve measurement stability. Tissue birefringence changes will be quantitatively analyzed instead of using the disappearance of colored bands in net retardance images as a qualitative criterion. We anticipate that real-time guidance with PSOCT may eventually reduce the recurrence of AF after PVI, thus giving patients a more robust curative option for treatment.

Videos are provided as supplemental material to show the progression over time of transmural and overtreated lesions monitored with the integrated PSOCT-RFA catheter, corresponding to the examples shown in Figs. 2 and 3. Please click on Video 1 and Video 2 to download and watch the videos.
